# Egg Quality during the Pubertal Transition—Is Youth All It’s Cracked Up to Be?

**DOI:** 10.3389/fendo.2017.00226

**Published:** 2017-09-04

**Authors:** Francesca E. Duncan

**Affiliations:** ^1^Department of Obstetrics and Gynecology, Northwestern University, Chicago, IL, United States

**Keywords:** egg, puberty, aneuploidy, fertility preservation, *in vitro* maturation, ovarian tissue cryopreservation, adolescent sterility, adolescent subfecundity

## Abstract

Although it is well accepted that egg quality decreases with advanced maternal age, we do not know how it is affected at the earliest ages during the pubertal transition—likely because this young population is not typically reproducing. However, in the setting of fertility preservation, more childhood cancer patients are surviving their diagnosis due to medical advances, forcing patients and their families to consider their future fertility at a very young age. *Ex vivo in vitro* maturation, in which cumulus oocyte complexes harvested from ovarian tissue are cultured to obtain mature gametes, is gaining traction as a fertility preservation method that is coupled to ovarian tissue cryopreservation. This method is particularly suitable for prepubertal and young adolescent girls, although live births have not yet been reported in gametes derived from females during the pubertal transition. Importantly, the period immediately following menarche in primate species (non-human primate and human) is characterized by relative subfecundity or sterility, and data from agricultural species and humans suggest that this may in part be due to increased chromosomal abnormalities in the egg. Together these data provide a compelling rationale for pushing the age boundary of when egg quality is considered, for performing further basic research to understand egg quality during this period, and for appropriately counseling patients.

Egg quality is intimately linked to female age. Aneuploidy—or the incorrect chromosome number of a cell—increases in the egg with advanced reproductive age (1). For example, trisomic conceptions are estimated to occur in ~2% of women in their twenties but increases to ~35% in women in their early forties (2). The age-associated decrease in egg quality is a leading cause of infertility, miscarriages, and birth defects (3, 4). These consequences of reproductive aging are becoming a significant concern as more women worldwide are delaying childbearing (5). Although egg quality decreases the farther females progress on the age spectrum, how it is affected at the other age extreme of youth is not well understood. This is largely because conception at very young ages is typically discouraged, especially in developed countries. However, modern trends in assisted reproductive technologies (ART) are fundamentally changing the landscape of how we reproduce, resulting in an urgent and unmet need to examine egg quality even in the youngest females. This concept is perhaps most tangible in the setting of oncofertility—or fertility preservation for cancer patients.

## The Field of Oncofertility Necessitates Understanding Egg Quality in Young Females

In the United States, childhood cancer rates have been increasing, and in 2016, approximately 10,400 children younger than 15 years were diagnosed with cancer.^1^ Due to advances and improvements in life-preserving treatments, >80% of childhood cancer patients will survive their diagnosis for at least 5 years (6). Moreover, the reduction in late effects of treatment exposure means that children and adolescents who were successfully treated for cancer will likely experience a longer lifespan and decreased late mortality (6). Nevertheless, cancer survivors have an elevated risk of severe, disabling, life-threatening, or fatal health conditions that span well into adulthood relative to healthy sibling counterparts (7). For example, chemotherapy and radiotherapy can negatively impact all aspects of the female reproductive system and lead to an array of adverse reproductive outcomes, such as follicular loss, endocrine abnormalities, and uterine dysfunction (8, 9). To counteract these unintended but potentially devastating consequences of cancer treatments on reproductive function, females may elect to preserve their fertility.

To date, standard fertility preservation methods include egg and embryo freezing. These methods, however, are contraindicated or not feasible in prepubertal girls. Instead, the most widely used fertility preservation option for prepubertal girls is ovarian tissue cryopreservation (OTC) (10). In this procedure, ovarian tissue—either a cortical biopsy or whole organ—is removed, processed, and cryopreserved. Following cancer treatment, ovarian tissue can be thawed and transplanted back into the individual to restore endocrine function and/or fertility (11). OTC followed by transplantation is successful, having resulted in nearly 100 live births globally (12). Moreover, this technology has been used to induce puberty in girls who suffered from premature primary ovarian insufficiency due to gonadotoxic treatments (13, 14). In 2016, the first live birth was reported in the popular press following transplant using tissue that was cryopreserved in a prepubertal girl. In this case, a 24-year-old beta thalassemia patient gave birth using transplanted tissue that she had frozen when she was only 9 years old.^2^

*Ex vivo in vitro* maturation (IVM) is a relatively new concept that has been coupled to OTC to expand fertility preservation options for females including prepubertal girls (15). When ovarian tissue is processed for OTC, thin strips of cortical tissue containing primordial follicles are prepared and cryopreserved. The ovarian medulla, which contains small antral follicles, is currently not cryopreserved. Instead, cumulus oocyte complexes (COCs) can be isolated from small antral follicles within the medulla, and IVM can be performed to obtain mature metaphase II-arrested eggs that can be cryopreserved or fertilized (16, 17). Although the efficiency is low, *ex vivo* IVM has been successful, with pregnancies and live births reported in cancer survivors (18–20). Mature gametes can be generated by *ex vivo* IVM even in prepubertal patients as young as 5 years old (21). Pregnancies and live births, however, have not yet been reported from eggs derived from *ex vivo* IVM in prepubertal girls. With the use of OTC steadily rising in young females younger than 18, we are forced to reconsider egg quality at a new extreme (22, 23). Just because we are clinically able to obtain mature gametes from a prepubertal population that is not normally capable of reproducing physiologically, should we? Data in humans and animal models would suggest that we need to carefully consider egg quality in the youngest females.

## Human and Animal Data Suggest that Egg Quality is Compromised During the Pubertal Transition

For almost a century, it has been recognized that following menarche, girls experience a period of 1–3 years of adolescent sterility or subfecundity (24, 25). This was first appreciated from ethnology studies demonstrating that pregnancy was rare in various native populations around the time of puberty, and there is further support of this notion in numerous other groups (24, 25). For example, in the !Kung hunter-gatherer society in Kalahari, a period of adolescent sterility was documented between puberty (16.6 years old) and first birth (18.7 years old) (26). In Bangladesh, in a particular population where there was no contraception and marriage was close to the time of menarche, there was a delay in birth of up to 3 years in 40% of the couples (27). Although this population was undernourished, it is important to note that roughly 95% of the couples eventually sired offspring during their reproductive lifespan (27). As Dr. Carl Hartman eloquently stated: “The onset of menstruation in girls is, of course, a momentous event. Nevertheless, though the mores of a given people may force ‘effective marriage’ upon them at this moment, there is much indication that, by and large, nature herself prevents motherhood supervening during an important series of preparatory years” (24).

Studies in animal models have further confirmed this phenomenon of adolescent sterility. For example, early studies in mice demonstrated that in the first estrous cycle, only 24% of matings resulted in pregnancies compared to 80–90% in 3- to 6-month-old mice (28). From these data, a ~30-day interval in which a majority of animals were incapable of conceiving and were effectively sterile was inferred. Adolescent sterility has also been documented in non-human primates (24, 25, 29). For example, in rhesus macaques, pubertal changes including menstruation, sexual swellings, and first ovulations begin when animals are ~2.5 years old, but the average age of first parturition ranges between 3 and 6 years (29). Similar periods of adolescent sterility have also been documented in the orangutan, chimpanzee, and gorilla (26). Thus, the interval between menarche and the ability to reproduce appears to be a normal progression of development among mammals. Potential reasons for this period of sterility have been proposed and include inadequate nutrition, insufficient body fat, anovulatory menstrual cycles, ovulatory menstrual cycles without sufficient luteal phases, and/or lack of coordination between sexual behavior and ovulation (26, 29).

Diminished egg quality also appears to be characteristic of early ovulatory cycles and may be an important factor contributing to suboptimal reproductive outcomes and adolescent sterility. In fact, several studies in human indicate that egg aneuploidy levels are higher in young females. For example, trisomy 21, which leads to Down syndrome, is tightly correlated to maternal age irrespective of geographic location, ethnicity, or socioeconomic status (2). The origins of trisomy 21 are largely due to errors arising during meiosis in the egg. Interestingly, there are several reports of increased trends of Down syndrome among extremely young mothers, but these were conducted in small populations and were not always significant (2).

The notion that aneuploidy is elevated in young females is further corroborated by a large retrospective review of comprehensive chromosomal screening (CCS) performed on 15,000+ trophectoderm biopsies to evaluate the impact of maternal age on aneuploidy prevalence (30). This study, which represents the largest systematic report of CCS in the general ART population, found that the lowest risk of having an aneuploid embryo was observed in women between 26 and 30 years. Both younger and older age groups were at risk of having higher incidence of aneuploidy and increased risk of complex aneuploidies. In fact, the incidence of aneuploidy was >40% in females younger than 23 years, and females in the youngest age cohort also had an increased likelihood of having no euploid embryos. Data from our own work support these observations (31). We performed IVM on COCs from ovarian tissue from females who had their ovaries removed for medical indications, and we evaluated aneuploidy in 16 eggs from 6 females ranging in age from 16 to 37 years. We observed an overall incidence of aneuploidy of 31%, and four of the five aneuploid eggs were from women older than 30 years. However, an egg from a 19-year-old woman was aneuploid and exhibited premature separation of sister chromatids (31). Although the sample size is limited in this study, it was done in an IVM model and, therefore, has important implications for the field of fertility preservation.

Decreased egg quality during the pubertal transition appears to be phylogenetically conserved, and this has been characterized in agricultural species where it is well known that sexually immature animals produce gametes of reduced developmental competence. For example, in the porcine model, a cytogenetic evaluation of eggs from gilts at the first estrus compared to third estrus demonstrated that the percent of immature oocytes (33.1 vs. 24.1%) and frequency of non-disjunction in mature oocytes was higher (21.6 vs. 11.9%) in the pubertal cohort (32). In addition, when IVM was performed using oocytes from prepubertal gilts relative to adult sows of the same breed, there was an eightfold increase in aneuploidy in eggs from the gilts (33). As research is increasing in this area, it is becoming clear that the differences in egg quality between the pubertal transition and sexual maturity are not limited to chromosomal abnormalities. Instead, the changes in egg quality are complex, with significant differences observed in gene expression patterns, cytoplasmic organelle composition and distribution, and follicular microenvironment (34–36).

## Redefining the Age Spectrum of Egg Quality

**Figure 1:**
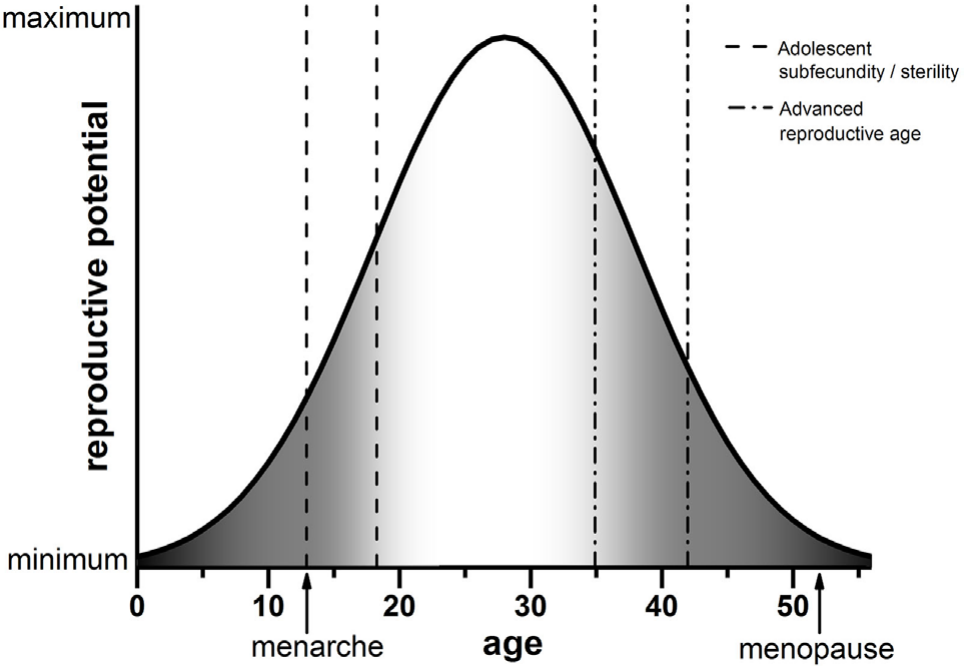
Schematic of reproductive potential and age. The reproductive potential of females changes significantly with age. Following menarche, there is a period of reduced reproductive potential referred to as adolescent subfecundity or adolescent sterility (dashed line). Reproductive potential then peaks when females are in their mid-twenties. A decline in reproductive potential begins when women reach their mid-30s, which is considered advanced reproductive age (dashed-dotted line). Reproductive function ceases completely when females reach menopause. The period of reproductive aging is concomitant with a decline in egg quality, indicated by the gray shading (right). We posit that the developmental window of adolescent subfecundity or adolescent sterility is also characterized by decreased egg quality (gray shading, left). Note that the ages in this schematic are estimations used for illustrative purposes.

The advances in reproductive science and medicine, ranging from contraception to ART, have provided us with exquisite control over our reproduction. The frontiers that await us are no less exciting, including prospects of ovarian bioprosthetics and stem cell-derived gametes (37–39). As we move toward new and enabling modes of reproduction, we must carefully consider age as a variable. Although advanced reproductive age is undoubtedly associated with poor egg quality and reproductive outcomes, data from human and animal systems strongly suggest that the other end of the youth spectrum is not necessarily much better. This new conceptual framework of decreased egg quality both during early adolescence and advanced reproductive age is shown in Figure 1. In the setting of oncofertility where younger and younger—even prepubertal—females are storing ovarian tissue for future fertility, we must appropriately counsel patients of the unknowns. Moreover, we must perform rigorous and comprehensive research to understand the mechanisms that influence gamete quality at the age extremes. It is tempting to speculate that the endocrine instability that characterizes both the pubertal and menopausal transition may have a profound impact on how the egg develops.

## Author Contributions

FD performed the research and writing related to this manuscript.

## Conflict of Interest Statement

The author declares that the research was conducted in the absence of any commercial or financial relationships that could be construed as a potential conflict of interest.

## Footnotes

^1^www.cancer.org.

^2^http://www.cnn.com/2016/12/15/health/first-birth-from-frozen-ovarian-tissue/index.html.
